# Supplementary material for: Templated synthesis of cubic crystalline single networks having large open-space lattices by polymer cubosomes
Source: Nat Commun. 2018 Dec 14;9:5327. doi: 10.1038/s41467-018-07793-8 (PMC6293999; doi:10.1038/s41467-018-07793-8)
Supplement: Supplementary file 1 — Supplementary Information [file 41467_2018_7793_MOESM1_ESM.pdf]

Supplementary Information for

Templated synthesis of cubic crystalline single networks having large open-space lattices by polymer cubosomes

Y. La, J. Song et al.

Supplementary Methods.

All reagents and chemicals were used as received from Sigma Aldrich and TCI. Dichloromethane (MC) was distilled over CaH_2. Tetrahydrofuran (THF) was refluxed over a mixture of Na and benzophenone under N_2 and distilled before use. Unless otherwise noted, all reactions were performed under N_2. NMR spectra were recorded on an Agilent 400-MR DD2 magnetic resonance system and Varian/Oxford As-500 using CD_2Cl_2 and $CDCl_3$ as solvents. Molecular weight of block copolymers was measured on a Agilent 1260 infinity gel permeation chromatography (GPC) system equipped with a PL gel 5 μm mixed D column and differential refractive index detectors. THF was used as an eluent with a flow rate of 1 mL min^{-1} at 35 °C. A PS standard kit (Agilent Technologies) was used for calibration. Matrix-assisted laser desoption ionization time-of-flight mass spectroscopy (MALDI-TOF-MS) was performed on a Bruker Ultraflex II TOF-TOF mass spectrometer equipped with a nitrogen laser (335 nm). The analytical sample was prepared by mixing a THF solution of analyte with a THF solution of matrix (sinapinic acid).

Optical micrographs of inorganic replicas were measured on a OLYMPUS BX53M microscope using HAWK-30MU CCD camera. Reflectance spectrum of the polymer cubosomes (PCs) and inorganic replicas were measured on a CRAIC 20/20 PV microspectrophotometer in 200–800 nm spectral region (5.5 × 5.5 μm^2 aperture size). A drop of the sample solution was placed on a Quartz substrate and then dried overnight. A white standard aluminum mirror served as the reference.

Scanning electron microscopy (SEM) was performed on a Hitachi S-4300 at an acceleration voltage of 15 kV. The dried sample was placed on a conductive carbon tape or silver paste and then coated with Pt with a thickness of 3 nm by using Hitachi E-1030 ion sputter. Transmission electron microscopy (TEM) was performed on JEOL JEM-2100 microscope at 200 kV. Sample specimens were prepared by placing a drop of the sample solution on a carbon-coated Cu grid (200 mesh, EM science). The grid was air-dried overnight.

Transmission electron microscopy tomography (TEMT) images were obtained by JEM-1400 operating at 120 kV. The current density of electron beam remained constantly at 18 pA cm^{-2}. The tilt series of TEM images were recorded with a 1 s exposure time using a 2048 × 2048 pixel Veleta CCD camera (Olympus Softer Imaging Solutions). The pixel size of TEM images was calculated to be 1.42 nm (for × 50 K). The series of projection images for PC of PEG5503-PS150 and PEG5503-PS168 were acquired at holder tilt angles between –66° to 70° and –54° to 56°, with an increment of 2°. Alignment and reconstruction of the tilt series were carried out in IMOD using weighted bank-projection method. Subsequently data were denoised by nonlinear aniosotropic diffusion prior to visualization. The 3D volume was visualized in UCSF Chimera for generating 2D projections and recording the movie.

Synchrotron small angle X-ray scattering (SAXS) data were obtained on the 6D and 9A SAXS beam line at Pohang acceleration laboratory in Korea (PLS-II, 3.0 GeV). The sample-to-detector distance (SDD) was 3.5 m and 6.5 m, respectively. The concentrated suspension of the PCs was dried for 24 h in a freeze-dryer. Ti-SBA-15 was used as standard sample and scattering spectra of powder samples were taken in a transmission mode at room temperature (11.6 keV).

Fluorescence microscopy images of PCs in which fluorescein sodium salt (Sigma Aldrich, λ_{Ex} = 460 nm, λ_{Em} = 515 nm) is encapsulated were obtained using Leica SP8 X Confocal Microscope and Nikon NSIM Super-Resolution Microscope System. 5–10 μL of concentrated suspension of the fluorescently labeled cubosomes was placed on a slide glass and sealed with cover glass. Residual solution was removed by filter paper and cover glass was fixed with sealant.

The particle size of PCs was measured by analyzing SEM images of PCs. One hundred particles were selected for the image analysis from SEM images

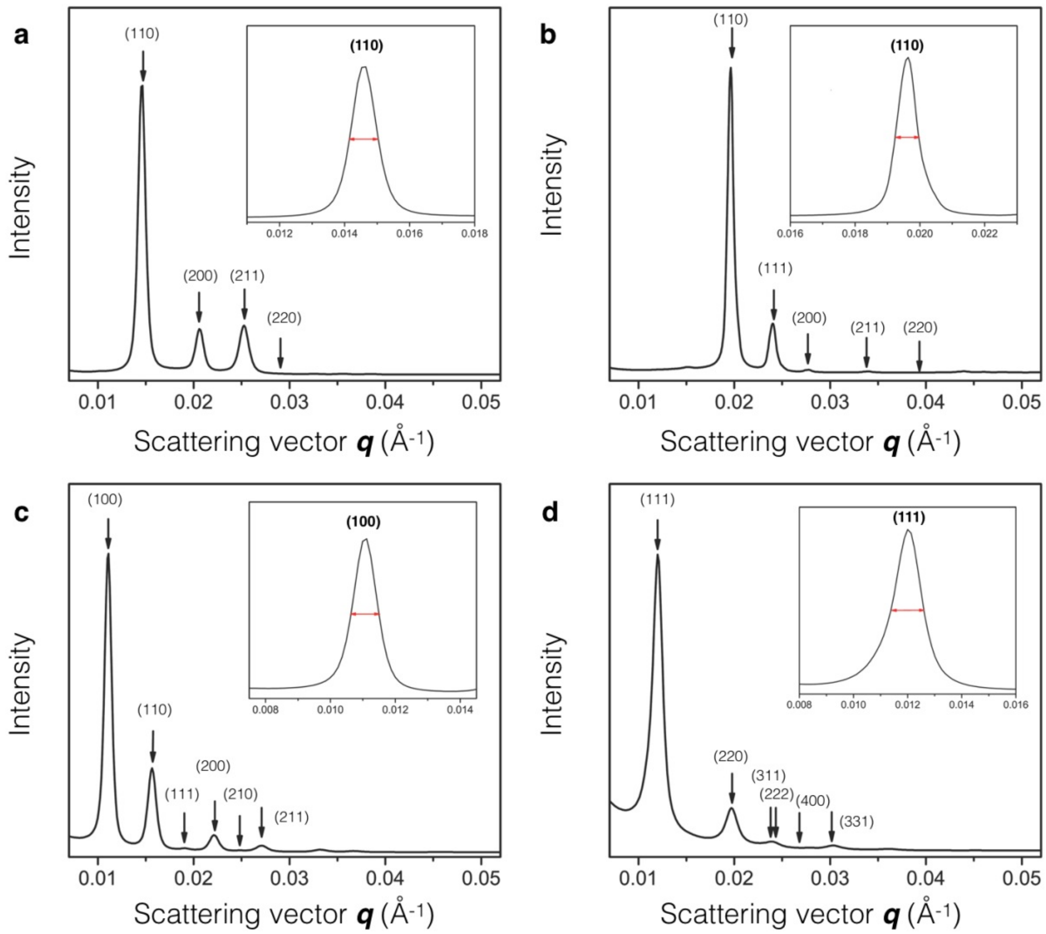

Supplementary Figure 1. SAXS results of PCs and silica replicas. a,b, PCs of PEG550$_3$-PS$_{150}$ ($Im\bar{3}m$, a = 60.7 nm) (a) and PEG550$_3$-PS$_{168}$ ($Pn\bar{3}m$, a = 45.2 nm) (b). **c,d,** silica replicas of PEG550$_3$-PS$_{150}$ ($Pm\bar{3}m$, a = 56.5 nm) (c) and PEG550$_3$-PS$_{168}$ ($Fd\bar{3}m$, a = 90.7 nm) (d). The insets show first diffraction peak position. Double headed arrows indicate the full width at half-maximum of the peak (Δq = 9.34 × 10^{-4} Å^{-1} (a), 8.78 × 10^{-4} Å^{-1} (b), 7.41 × 10^{-4} Å^{-1} (c), 1.22 × 10^{-3} Å^{-1} (d)). The size of crystallites was estimated as 673 nm for the internal Schwarz P surface of PEG550$_3$-PS$_{150}$ and 716 nm for its replica. For the Schwarz D surface of PEG550$_3$-PS$_{168}$, the size of crystallite was estimated as 833 nm and 516 nm for its single diamond replica.

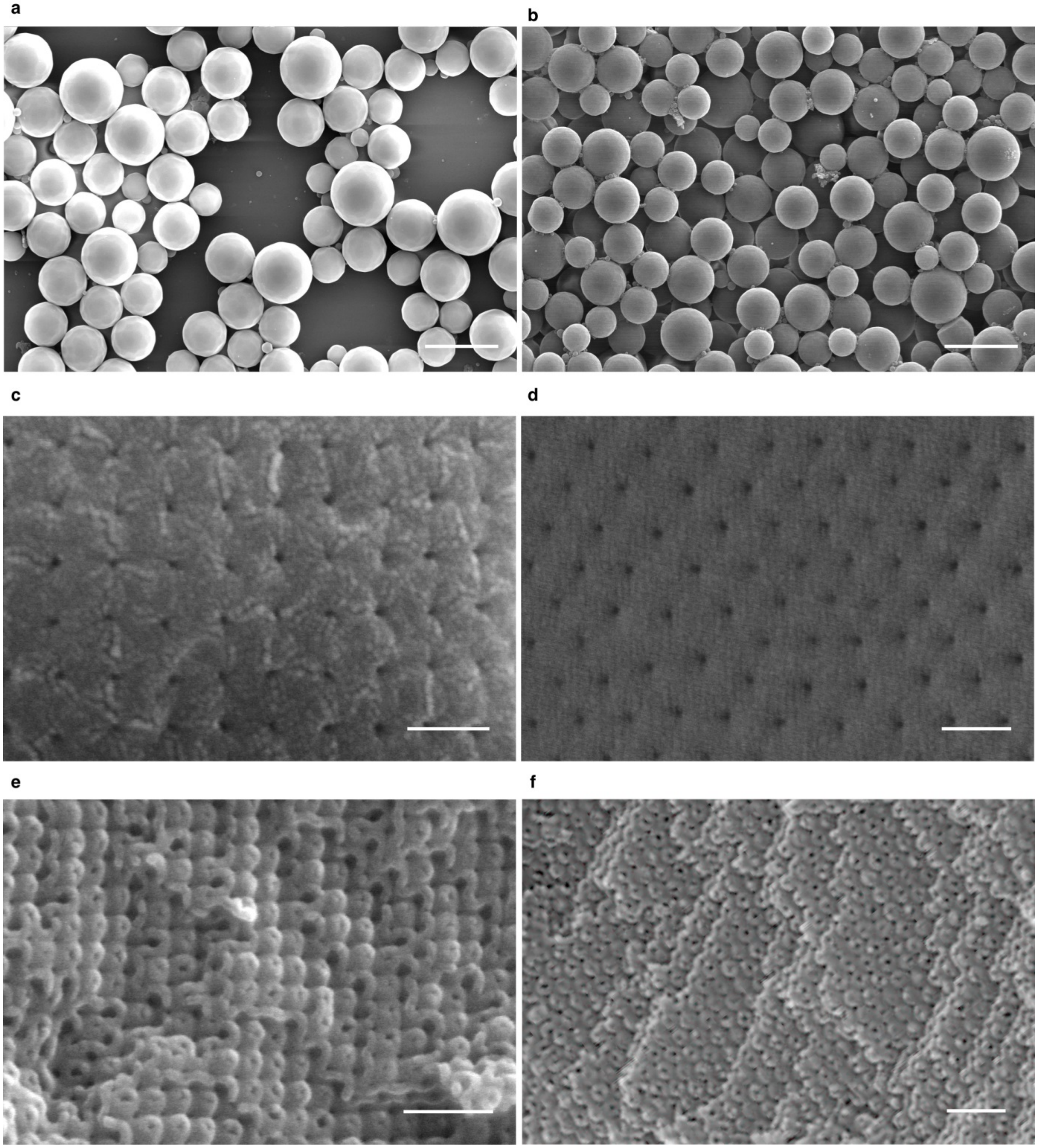

Supplementary Figure 2. SEM images of PCs. a,b, Low-magnification SEM images of PCs of PEG550$_3$-PS$_{150}$ (a) and PEG550$_3$-PS$_{168}$ (b). Scale bars, 20 μm (a) and 30 μm (b). **c,d,** SEM images showing surface of the PCs of PEG550$_3$-PS$_{150}$ (Schwarz P surface) (**c**) and PEG550$_3$-PS$_{168}$ (Schwarz D surface) (**d**). Scale bars, 100 nm. **e,f,** SEM images of internal structures of PCs of PEG550$_3$-PS$_{150}$ (**e**) and PEG550$_3$-PS$_{168}$ (**f**). Scale bars, 200 nm

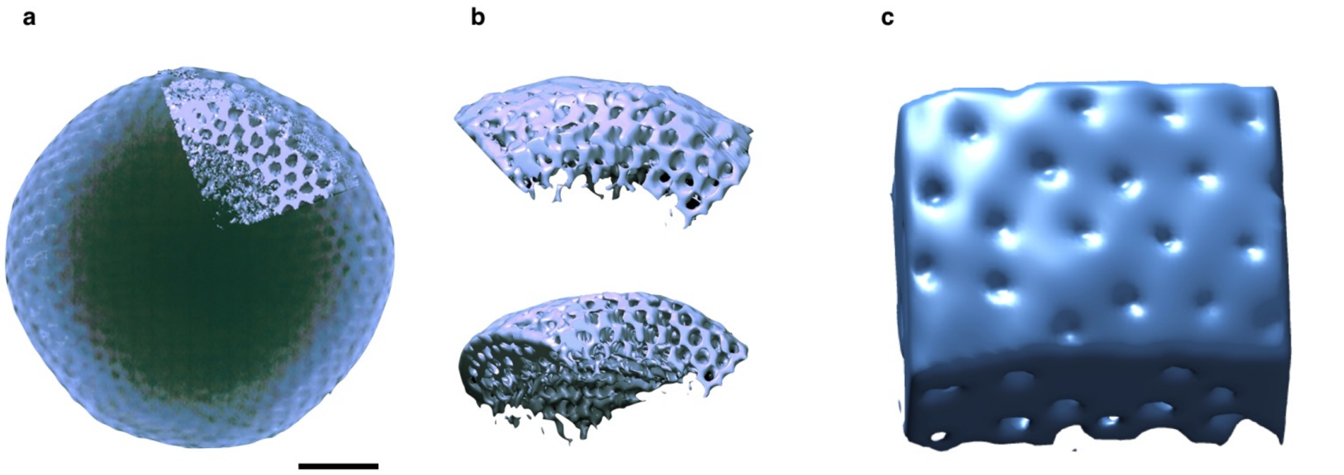

Supplementary Figure 3. TEM tomograms of PCs of $PEG550_3$-PS_{168}. a, 3-D reconstructed TEM image of the PCs. Scale bar, 200 nm. **b,** Front and tilted cross-sectional view of surface side of PCs. **c,** Top view of the reconstructed TEM image showing hexagonally arranged porous surface.

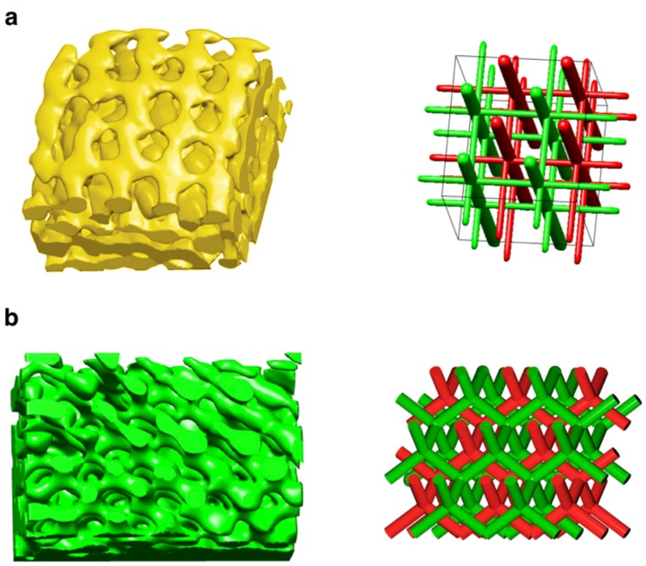

Supplementary Figure 4. 3-D reconstructed TEM tomograms and computer-generated projection of the internal channel networks of PCs. a, Internal networks of PCs of PEG550$_3$-PS$_{150}$ showing primitive cubic networks with six-fold nodes. The lattice diagram at the right indicates the skeletal networks of $Im\bar{3}m$ space group. **b,** Internal networks of PCs of PEG550$_3$-PS$_{168}$ showing the diamond lattice with four-fold nodes. The lattice diagram at the right indicates the skeletal networks of $Pn\bar{3}m$ space group.

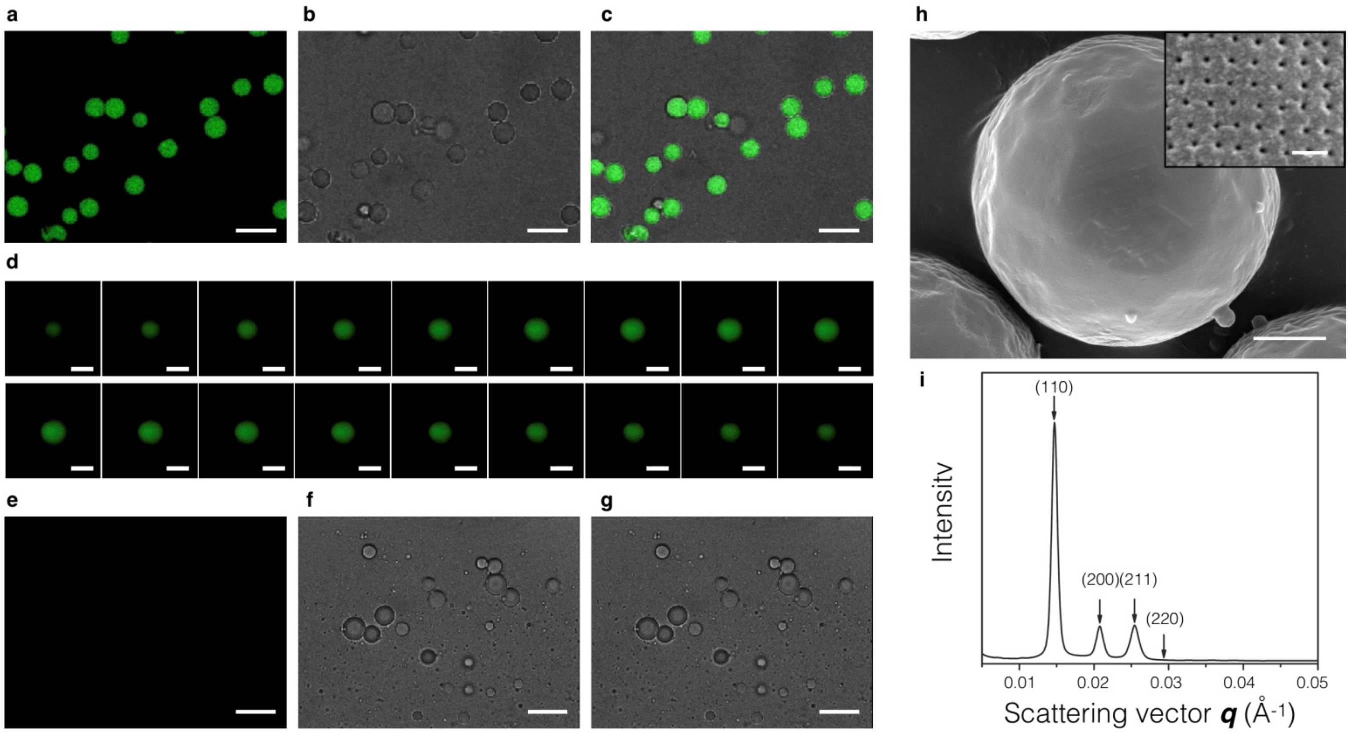

Supplementary Figure 5. CLSM and SIM images of the PCs of PEG550$_3$-PS$_{150}$. a,b,c, CLSM images of PCs self-assembled in the presence of Fluorescein in water (λ_{Ex} = 460 nm, λ_{Em} = 515 nm) : (a) dark field, (b) bright field, (c) merged. The merged image showed the presence of dye in the water channels in PCs. Scale bars, 10 μm. **d,** SIM images obtained from different focal planes (interval of z-direction : 100 nm). Scale bars, 2 μm. **e,f,g,** CLSM images of PCs mixed with fluorescein in water after purification : (e) dark field, (f) bright field, (g) merged. The dark field and merged images did not show any retention of dye molecules. Scale bars, 20 μm**. h,** SEM images of fluorescein-encapsulating PCs. Scale bar, 2 μm. The inset shows magnified view of perforated surface of colloidal PC. Scale bar, 100 nm. **i,** SAXS result of fluorescein-encapsulating PCs shows that the phase and lattice constant are similar to PCs formed without fluorescein ($Im\bar{3}m$, a = 60.2 nm).

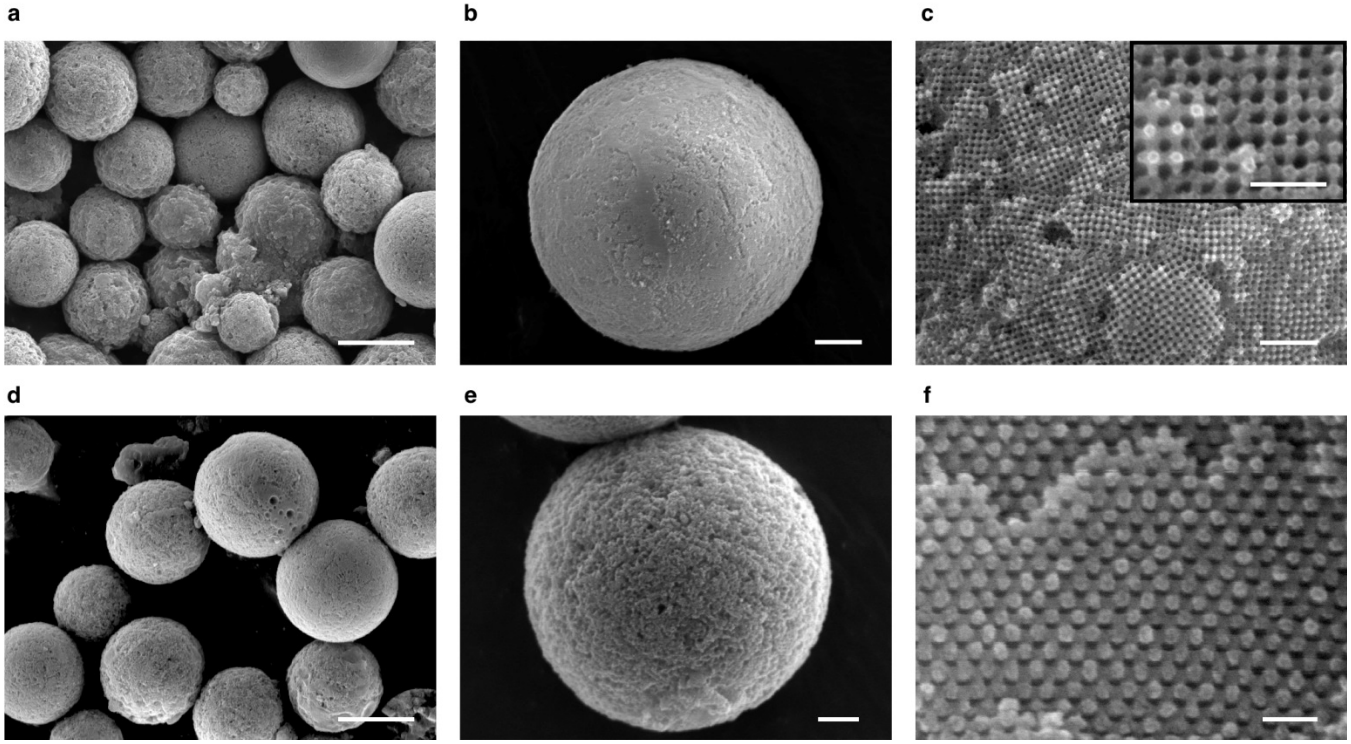

Supplementary Figure 6. SEM images of the silica replica of the PCs. a,b,d,e, SEM images showing spherical SiO_2 particles replicated from cubosome templates of PEG550$_3$-PS$_{150}$ (a,b) and PEG550$_3$-PS$_{168}$ (d,e). Scale bars, 10 μm (a,d) and 2 μm (b,e). **c,f,** High-magnification SEM images showing silica framework on the surface of the replicated structures of $Pm\bar{3}m$ (c) and $Fd\bar{3}m$ (f). Scale bars, 500 nm (c), 200 nm (c inset), and 100 nm (f).

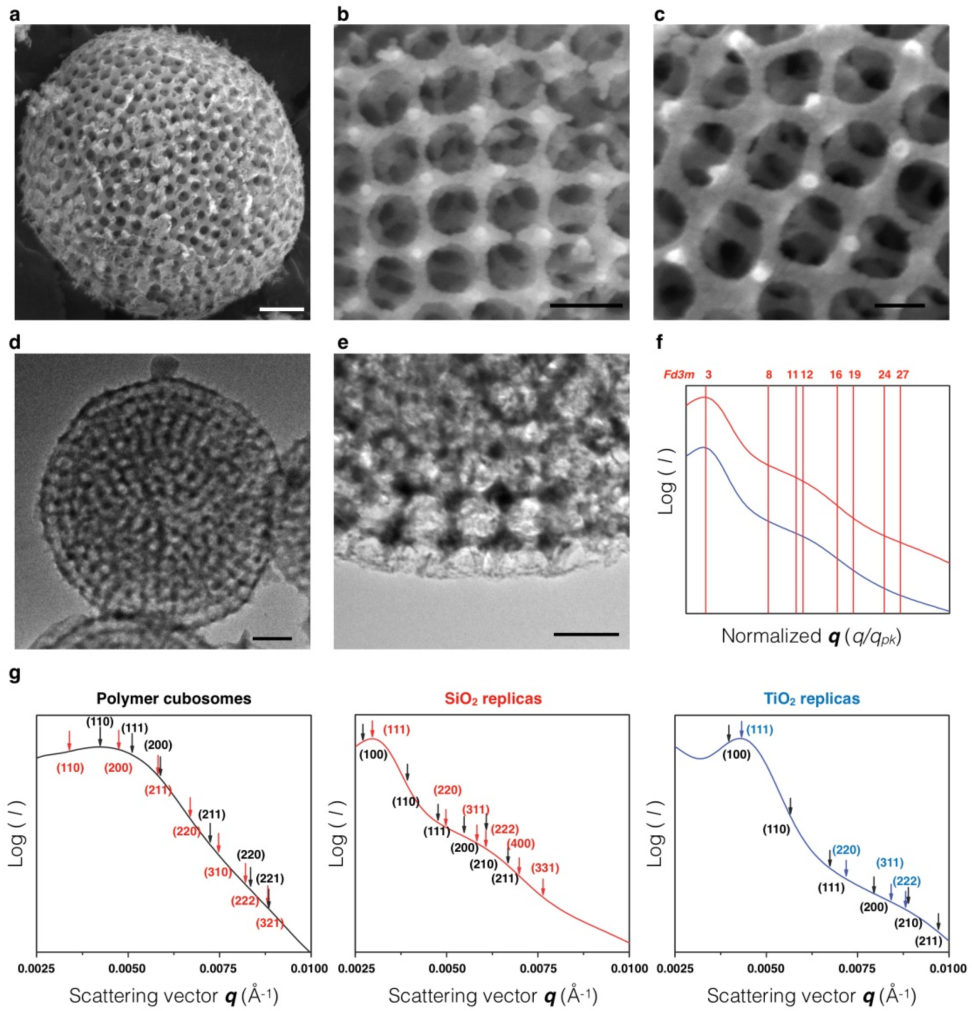

Supplementary Figure 7. Structural characterization of the titania replica of PCs of PEG2000$_3$-PS$_{2140}$. a, Low-magnification SEM image showing spherical replicas. Scale bar, 500 nm. **b,c,** SEM images of structures of the TiO_2 replicas showing $Pm\bar{3}m$ space group (b) and $Fd\bar{3}m$ space group (c). Scale bars, 200 nm (b) and 100 nm (c). **d,e,** TEM images showing the single network of replicas. Scale bars, 500 nm (d) and 200 nm (e). **f,** Normalized SAXS results of skeletal silica replicas (red line) and titania replicas (blue line). Vertical lines correspond to the expected Bragg peak positional ratios of $Fd\bar{3}m$ space group. **g,** SAXS results of the PCs and replicas assigned to mixed phases. The expected peak position of $Im\bar{3}m$ space group of PCs was calculated from the lattice parameter relationship of two phases ($a_{p\ surface}/a_{d\ surface}$ = 1.279). Polymer cubosomes; $Pn\bar{3}m$ (a = 213 nm, black) and $Im\bar{3}m$ (a = 273 nm, red). SiO_2 replicas; $Fd\bar{3}m$ (a = 361 nm, red) and $Pm\bar{3}m$ (a = 231 nm, black). TiO_2 replicas; $Fd\bar{3}m$ (a = 247 nm, blue) and $Pm\bar{3}m$ (a = 159 nm, black).

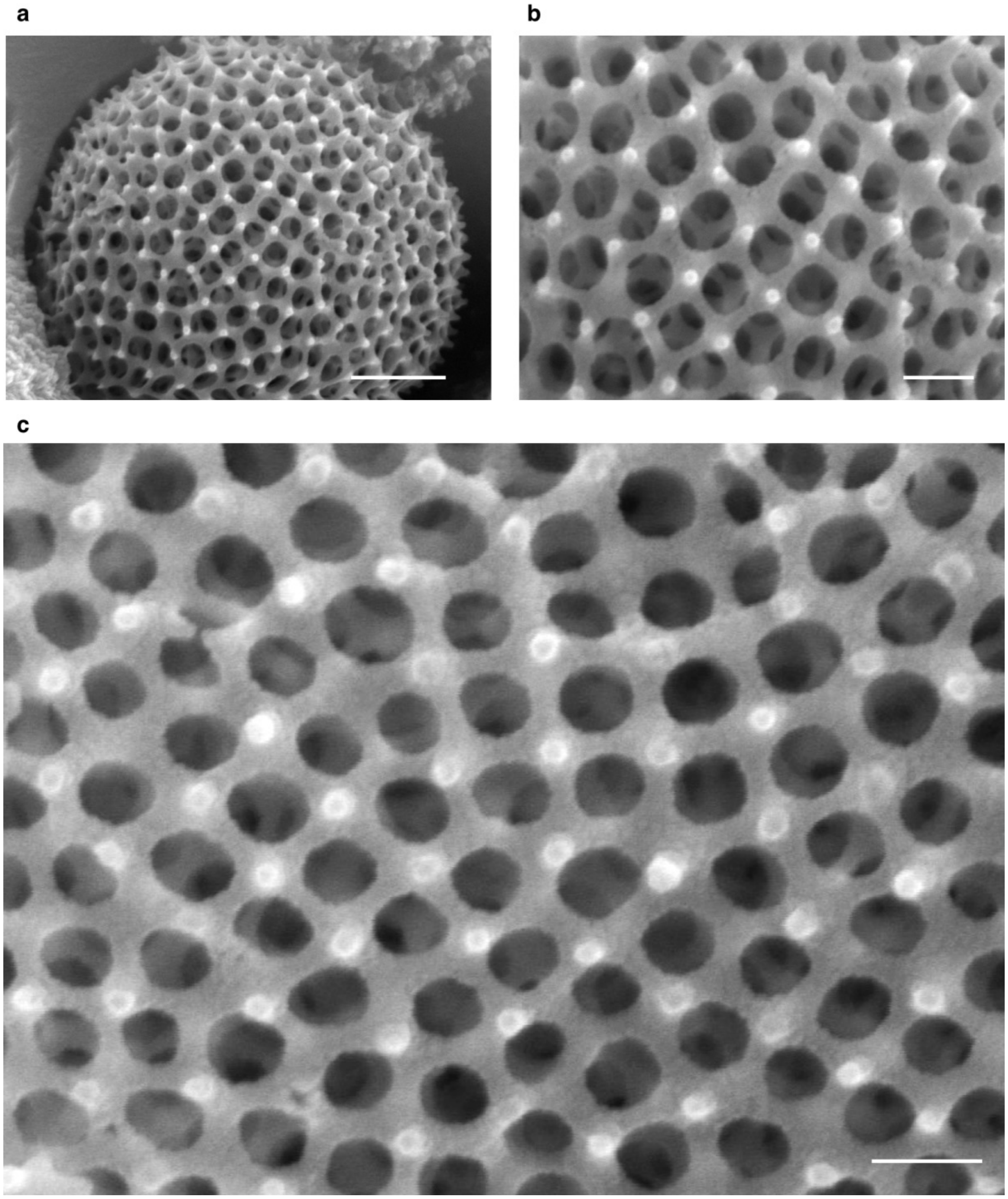

Supplementary Figure 8. SEM images of the SiO_2 replica of PCs of PEG2000$_3$-PS$_{2140}$. a,b, Low-magnification SEM image showing the single cubic network of the spherical replica (a) and magnified view of surfaces of the replica showing distorted lattice (b). Scale bars, 500 nm (a) and 200 nm (b). **c,** SEM image of the single cubic network of SiO_2 replicas showing $Pm\overline{3}m$ space group on the surfaces. Scale bar, 200 nm.

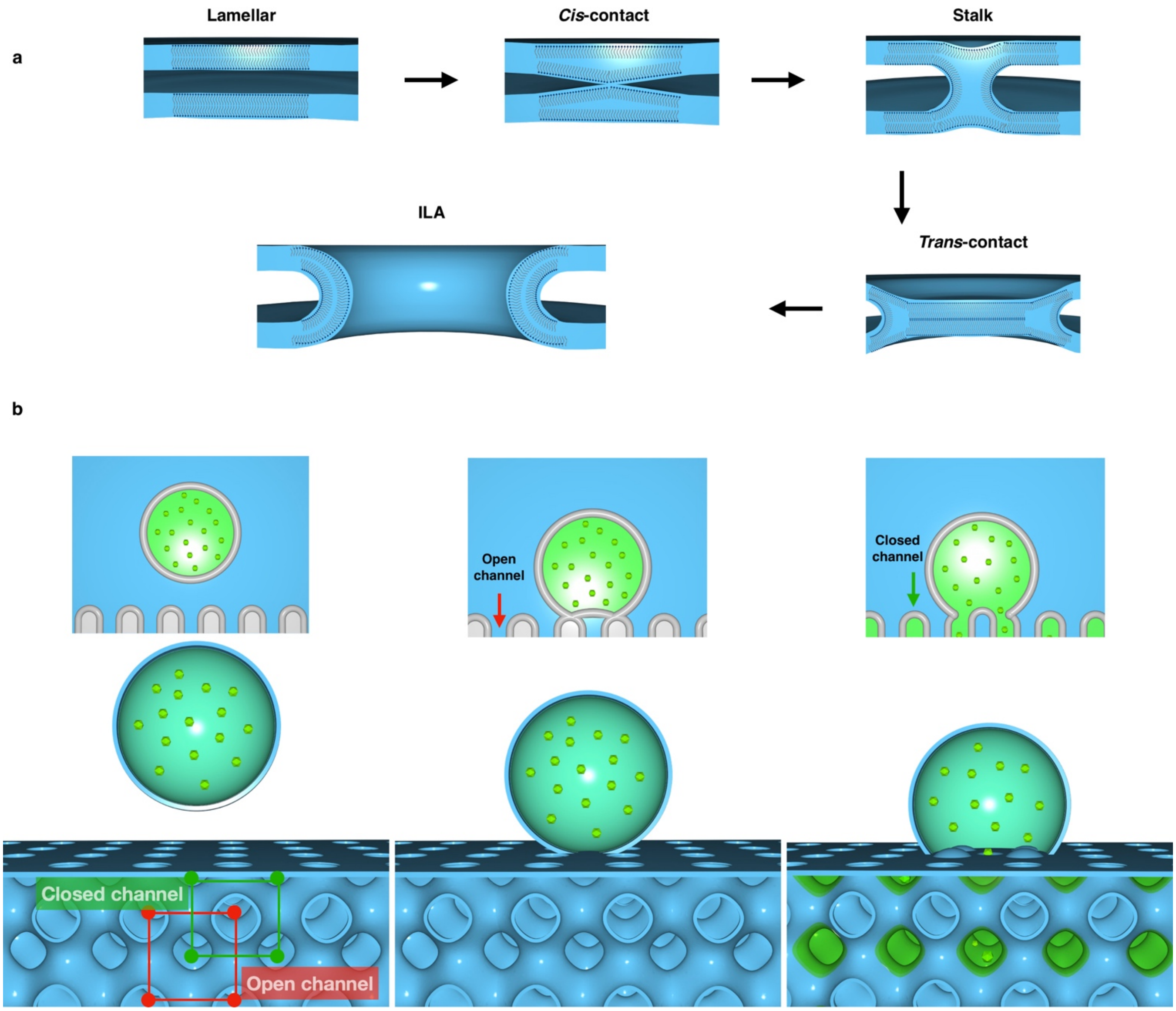

Supplementary Figure 9. A schematic representation of the topological inversion at the interface between membranes. a, Interlamellar attatchment (ILA). **b,** Polymersomes and PCs created by fusion. Polymer vesicles containing fluorescent molecules undergo fusion to the PC, resulting in the relocation of bilayer membranes to connect the lumen of a polymer vesicle to the closed channel embedded in a PC. Fluorescent molecules residing in the lumen of a polymer vesicle are confined within the closed channel of a PC because the inversion of the topology only occurs at the interface created by fusion. The open channel is closed at the interface.

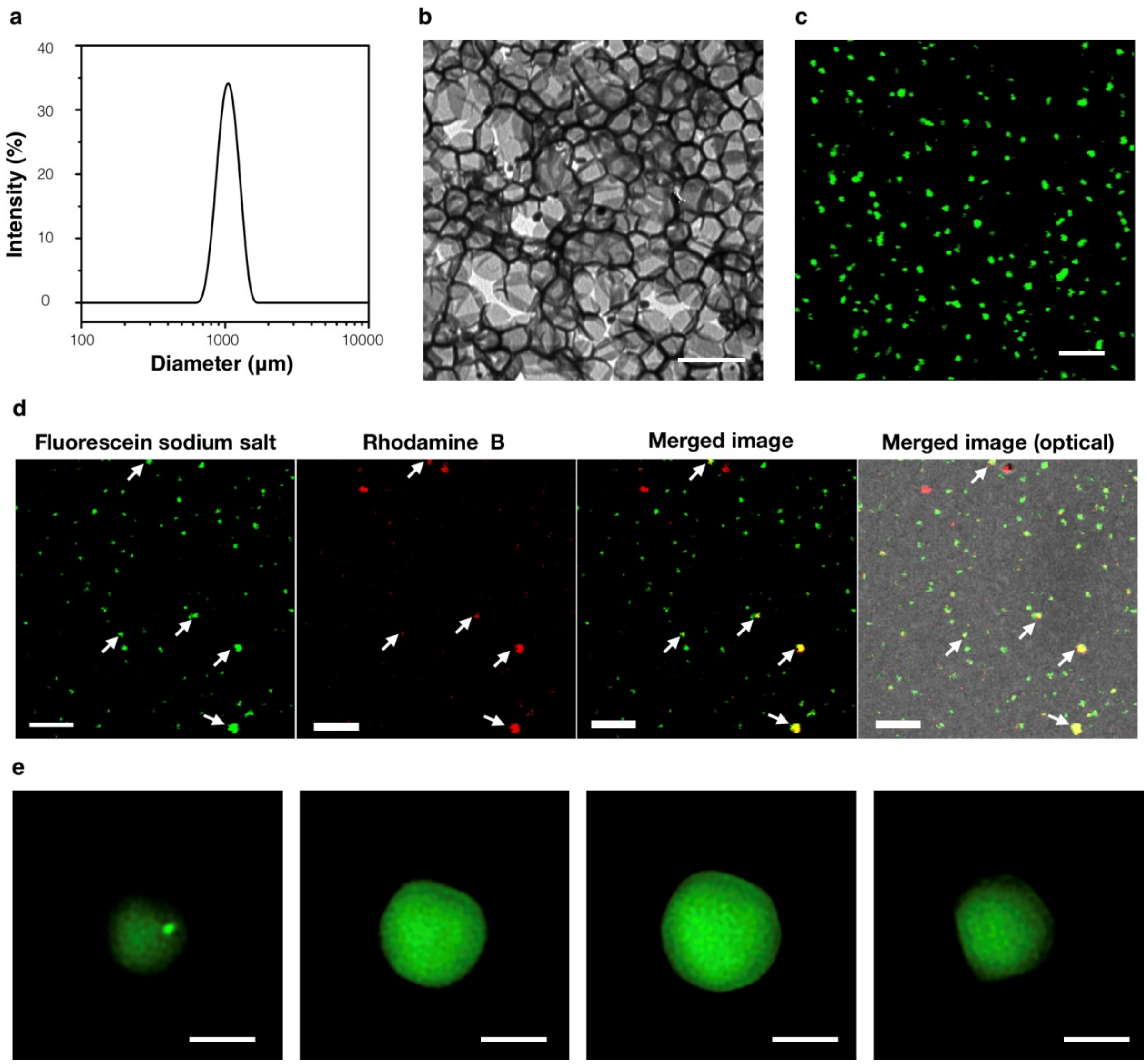

Supplementary Figure 10. Fluorescein-encapsulating polymersomes and their fusion with PCs. a,b, Size distributions (a) and TEM image (b) of the fluorescein-encapsulating polymersomes of PEG550$_3$-PS$_{120}$ (d = 992 nm). Scale bar, 2 μm. **c,** CLSM image of fluorescein-encapsulating polymersomes. Scale bar, 5 μm. **d,** CLSM images showing the fusion of fluorescein- and rhodamine B-encapsulating polymersomes. White arrows indicate fused polymersomes which exhibit orange color in merged images. Scale bars, 5 μm. **e,** SIM images of a fused PC obtained from different focal planes (interval of z direction : 800 nm). Scale bars, 2 μm.

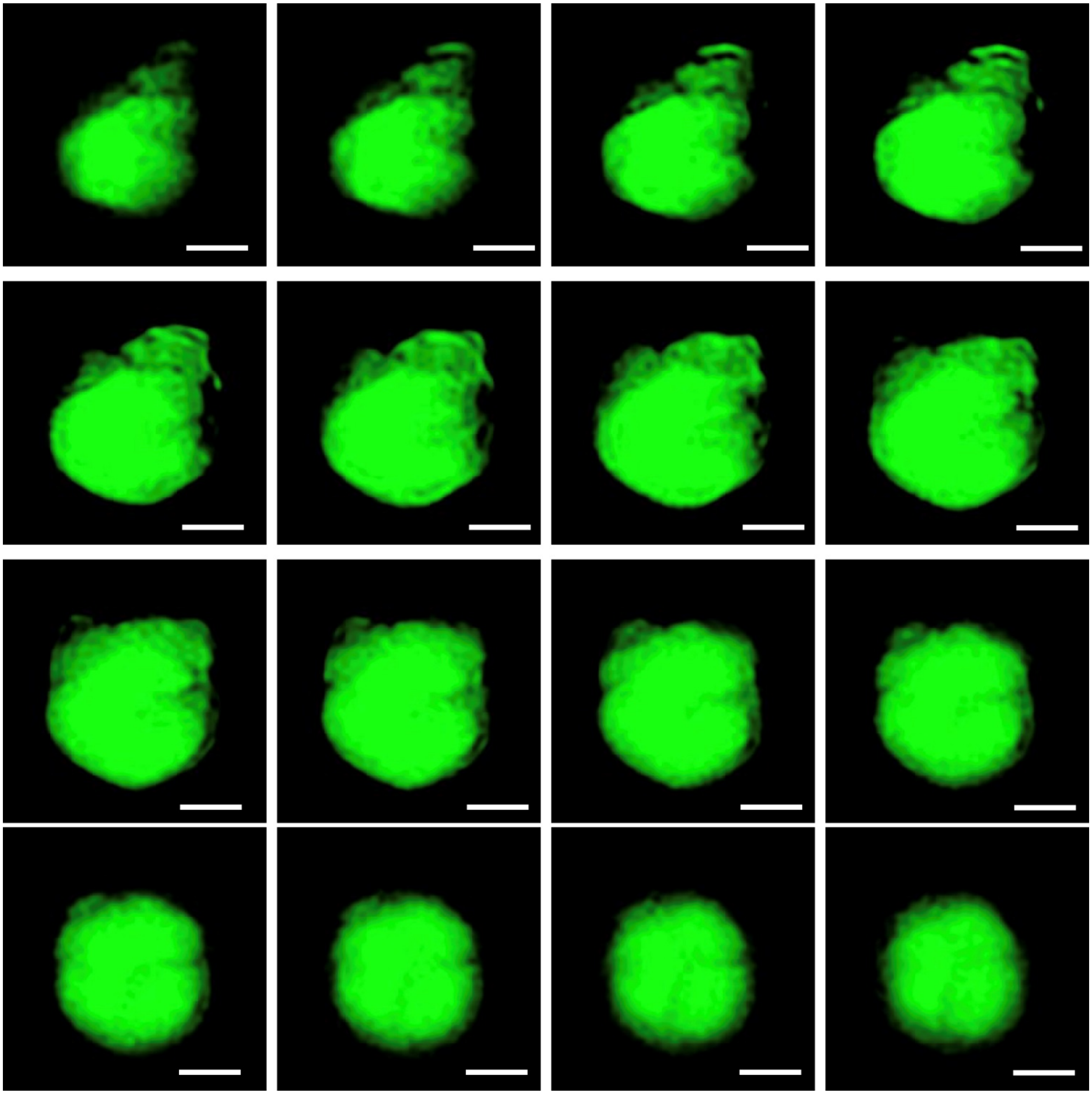

Supplementary Figure 11. Super-resolution fluorescence microscopy images of a PC fused with polymersomes. SIM images obtained from different focal planes (interval of z-direction: 200 nm). Scale bars, 1 μm.
