# Supplementary material for: Templated synthesis of cubic crystalline single networks having large open-space lattices by polymer cubosomes
Source: Nat Commun. 2018 Dec 14;9:5327. doi: 10.1038/s41467-018-07793-8 (PMC6293999; doi:10.1038/s41467-018-07793-8)
Supplement: Supplementary file 3 — Description of Additional Supplementary Files [file 41467_2018_7793_MOESM3_ESM.pdf]

**Description of Additional Supplementary Files**

File Name: Supplementary Movie 1

Description: TEM tomography of a PC of PEG5503-PS150. Left, recorded two-dimensional projections of PCs with different tilt angles. Right, tomography of the PC showing bicontinuous internal channel networks.
